# HBA-DEALS: accurate and simultaneous identification of differential expression and splicing using hierarchical Bayesian analysis

**DOI:** 10.1186/s13059-020-02072-6

**Published:** 2020-07-13

**Authors:** Guy Karlebach, Peter Hansen, Diogo FT Veiga, Robin Steinhaus, Daniel Danis, Sheng Li, Olga Anczukow, Peter N Robinson

**Affiliations:** 1 The Jackson Laboratory for Genomic Medicine, Farmington, 06032 CT USA; 2 Charité - Universitätsmedizin Berlin, Institute of Medical Genetics and Human Genetics, Berlin, 13353 Germany; 3 Berlin Institute of Health (BIH), Berlin, 10117 Germany; 4 Institute for Systems Genomics, University of Connecticut, Farmington, 06032 CT USA

**Keywords:** Differential expression, Alternative splicing, Transcription

## Abstract

We present Hierarchical Bayesian Analysis of Differential Expression and ALternative Splicing (HBA-DEALS), which simultaneously characterizes differential expression and splicing in cohorts. HBA-DEALS attains state of the art or better performance for both expression and splicing and allows genes to be characterized as having differential gene expression, differential alternative splicing, both, or neither. HBA-DEALS analysis of GTEx data demonstrated sets of genes that show predominant DGE or DAST across multiple tissue types. These sets have pervasive differences with respect to gene structure, function, membership in protein complexes, and promoter architecture.

## Background

RNA sequencing (RNA-seq) has become the most commonly used genomic technique for the transcriptome-wide analysis of differential expression and alternative splicing of mRNAs. Since its introduction over a decade ago, Illumina short-read sequencing technology has been the dominant platform for carrying out RNA-seq experiments, but newer long-read single-molecule sequencing technologies of Pacific Biosciences and Oxford Nanopore provide alternatives that may allow a more accurate and comprehensive assessment of isoform diversity [1]. Analysis of RNA-seq data is done in a pipeline that maps raw reads to genes or isoforms (transcripts), quantifies the number (count) of reads associated with each isoform, generating an expression matrix, followed by normalization steps and statistical analysis of differential expression [2, 3].

Algorithms for the analysis of differential gene expression or differential splicing have many different approaches. Differential gene expression (DGE) refers to alterations in the expression (counts) of the sum of each of the isoforms that are encoded by a gene. Many methods for DGE analysis are based on discrete probability distributions such as the Poisson or negative binomial [4, 5]. voom instead estimates the mean-variance relation non-parametrically from log-counts per million reads, which are used as input for linear modeling and empirical Bayes differential expression analysis [6].

In contrast to DGE, differential alternative splicing and transcription (DAST) refers to differential usage of isoforms that include distinct combinations of exons or begin from distinct transcription start sites. Computational methods for identifying DAST in RNA-Seq data can be broadly divided into two approaches. The first approach is based on an analysis of the percent spliced in (*Ψ*[Psi]), which is defined as $\frac {\text {IR}}{\text {IR}+\text {ER}}$, where IR refers to inclusion reads and ER to exclusion reads. This approach models differences in splicing as differences in *Ψ*, corresponding to the probability of an alternative splicing event at a splice junction [7–10]. A second approach compares counts of alternative isoforms [11–13].

Most existing methods look at either DGE or DAST but not both. When using separate procedures for differential splicing and expression, however, the determination of which genes are alternatively spliced, differentially expressed, undergo both of these changes, or none of them requires intersection between negative and positive findings. This is usually not possible without violating some of the assumptions of individual tests. For example, frequentist methods assess statistically significant differential expression or splicing using *p* values, but non-significant *p* values cannot be readily interpreted as providing evidence of lack of differential expression or splicing. Moreover, variation of gene expression can be affected by expression level [14], and similarly, variation of isoform expression is affected by isoform level. Methods that transform isoform levels into proportions (i.e., *Ψ*-based methods) do not model this relationship correctly, and therefore fail to accurately model dispersion, which is essential for determining significance. On the other hand, modeling individual isoform expression levels but not modeling their joint expression can result in false positives when a gene is differentially expressed but its isoforms are not differentially spliced, since a change in an individual isoform’s levels between conditions does not necessarily mean a change in that isoform’s proportion and vice versa.

In this work, in contrast, we present a Bayesian method for analyzing RNA-seq data that simultaneously identifies DGE and DAST based on isoform counts. We show with our method, using data from the Genotype-Tissue Expression (GTEx) project [15], that genes can be assigned to four groups according to the propensity of a gene to display DGE, DAST, both, or neither in comparisons between different tissue types. These classes differ not only with respect to gene functions and structure, but also with respect to the distribution of transcription factor binding sites (TFBS), membership in protein complexes, and methylation of their promoter regions.

## Results

Here, we present a method for joint modeling of differential expression and splicing, Hierarchical Bayesian Analysis of Differential Expression and ALternative Splicing (HBA-DEALS).

### A Bayesian model for simultaneous assessment of differential expression and splicing

**Fig. 1:**
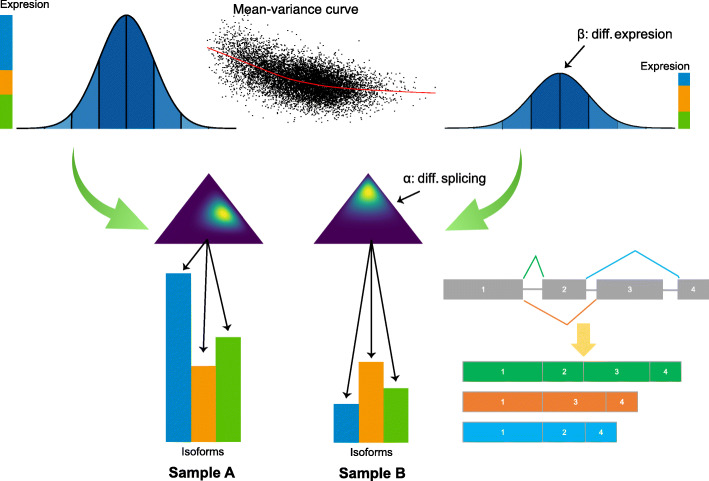
Overview of the HBA-DEALS model. The log-transformed expression of a gene with three isoforms (green, orange, and blue) is shown. The gene expression is the sum of the expression of the isoforms. Differential gene expression is modeled as two Normal distributions whose means differ by the parameter *β*. The proportions of the corresponding isoforms have a Dirichlet prior, and the difference in proportions between controls and cases is modeled by *α* (symbolized by the two triangles). An MCMC procedure is used to solve for the posterior distribution of the parameters of the model for all genes and isoforms at once. The marginal posterior distributions are in turn interpreted to classify each gene as DAST, DGE, both, or neither (the “Methods” section). In this example, the gene shows both downregulation at gene level and a change in the dominant isoform in sample B

HBA-DEALS is based on a hierarchical Bayesian model of the absolute expression levels of the gene and its isoforms (Fig. 1 and Additional file 1:Fig. S1). HBA-DEALS assumes that the data are available from *n* RNA-seq samples and that the sequence reads have been mapped to isoforms. The *n* samples are divided into two cohorts *n*_1_ and *n*_2_ (e.g., cases and controls). The output of any isoform quantification tool, including but not limited to Salmon [16], RSEM [17], Kallisto [18], and StringTie [19], can be used as the input for HBA-DEALS. Long-read isoform counts can be generated with pipelines such as SQANTI [20]. HBA-DEALS automatically sample-normalizes isoform counts.

The isoform counts are first log-transformed. The log gene expression levels are then modeled using a Normal distribution, with a mean that is equal to the log-transformed sum of corresponding mean isoform levels. A linear model is fit to each gene’s levels, and a trend line is then fit to the square root standard deviations as a function of mean gene level [6]. The variance in an individual sample is inferred from the corresponding value of the fitted trend line. The mean isoform expression is a fraction of the mean expression level of its gene (in Fig. 1, sample A and sample B display a different distribution of the three isoforms of an example gene), and similarly to gene expression, a sample-specific variance is obtained from a mean-variance trend for isoforms. The proportions of all isoforms assigned to a gene are represented as a vector of isoform fractions [*p*_1_,*p*_2_,...,*p*_*n*_] with $\sum p_{i}=1$ (isoform fractions are symbolized by the triangles in Fig. 1). The prior of the isoform fractions is Dirichlet distributed with the vector [1,1,...,1].

In order to model difference in gene expression, a parameter *β* is added to the mean expression level in one condition. A weakly informative $\mathcal {N}(0,5)$ prior is assigned to *β*. This prior represents our belief that the most common state of a gene is not differentially expressed, but large fold changes are not much less probable. Unlike differential expression, simple addition cannot model difference in splicing because the entries of the vector of isoform fractions must sum to 1, and addition does not preserve this property. Therefore, instead of adding a vector *α* to the isoform fractions in one condition, we apply an Aitchison perturbation [21] between *α* and the isoform fractions in that condition. Note that both *α* and *β* are parameters in a single hierarchical model, and therefore, HBA-DEALS estimates the posterior distributions of *α* and *β* simultaneously. A *β* value of 0 corresponds to a gene that is not differentially expressed. If the bulk of its marginal posterior distribution is entirely above or below zero, we interpret the gene to be differentially expressed; otherwise, the interpretation is that the gene is not differentially expressed (the “Methods” section). Similarly, an *α* vector in which all entries are equal corresponds to a gene that is not differentially spliced. For a gene with T isoforms, this corresponds to $\alpha =[\frac {1}{T},\frac {1}{T},...,\frac {1}{T}]$. We obtain the probability of differential splicing by examining the shifted posterior marginal probability for each isoform i, $P_{\alpha _{i}}(x-\frac {1}{T})$. If the bulk of this posterior distribution for some isoform is above or below zero, then we predict that the gene is differentially spliced (the “Methods” section).

The performance of HBA-DEALS is not overly sensitive to the parameters used in its weakly informative priors (Additional file 1: Fig. S2). In order to show that the MCMC chain is convergent, we applied Geweke’s convergence diagnostic to the MCMC runs on a complete simulated dataset using 5000 warmup steps and 5000 MCMC steps [22]. After multiple testing correction, none of the *p* values obtained using Geweke’s diagnostic were significant (Additional file 1: Fig. S3).

### Model validation

**Fig. 2:**
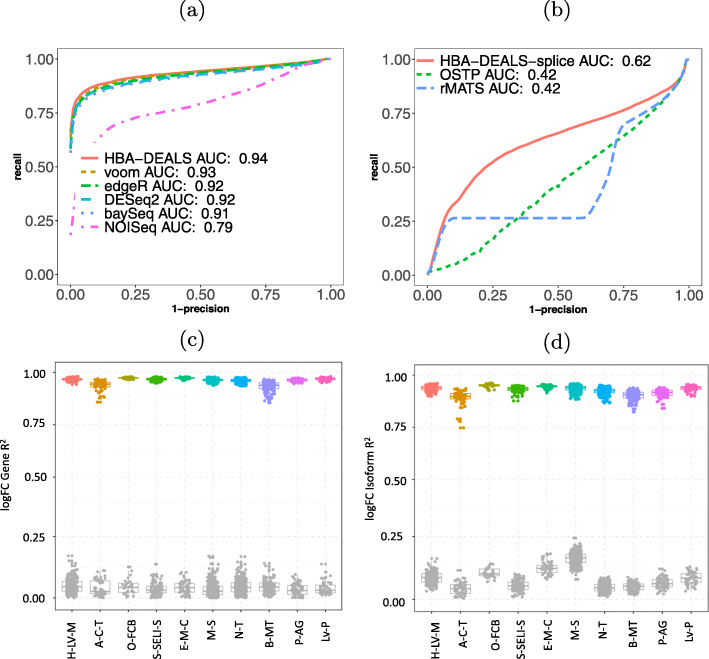
HBA-DEALS evaluation. **a** Precision-recall (PR) analysis of HBA-DEALS and five state-of-the-art algorithms for the detection of RNA-seq differential expression. **b** PR analysis of HBA-DEALS, OSTP, and rMATS. In panels **a** and b, the Y axis shows precision and the X axis shows 1-recall. **c***R*^2^ for gene expression (GTEx, distant). Data points shown in color represent correlations based on genes that were identified as differentially expressed by HBA-DEALS. As a control, genes not identified as differentially expressed with probability *p*≥0.25, see the “Methods” section) are shown in gray (abbreviations in Additional file 1: Table S2). **d***R*^2^ for isoform proportion (GTEx, distant). Data points shown in color represent correlations based on isoforms identified as differentially spliced by HBA-DEALS. As a control, isoforms from genes not identified as differentially spliced (*p*≥0.25) are shown in gray

We applied three approaches to assess the performance of HBA-DEALS. First, we extended an existing simulation scheme for RNA-seq expression data [6] to enable the modeling of alternative splicing. For each gene, we split its sample proportion between a random number of isoforms, and for differentially spliced genes, we doubled the proportion of one random isoform in cases and another in controls. We analyzed 50 simulated datasets for DGE with HBA-DEALS, voom [6], DESeq2 [5], edgeR [4], baySeq [23], and NOISeq [24]. We analyzed the same datasets for differential alternative splicing with HBA-DEALS, rMATS [8], and a method we call optimal splicing using the t-statistic and proportions (OSTP), which provides an upper bound on the performance of t-statistic-derived significance values to compare *Ψ* of isoforms (the “Methods” section). HBA-DEALS displayed a larger area under the precision-recall curve than the other approaches did for both DGE and DAST (Fig. 2a, b).

In order to assess the accuracy of HBA-DEALS using real data, we ran HBA-DEALS on estimated isoform levels from the Genotype-Tissue Expression project (GTEx) [15]. We used HBA-DEALS to identify differentially expressed and differentially spliced genes in 20 different pairs of tissues. We chose ten pairs of tissues that were closely related (e.g., subcutaneous adipose tissue and visceral adipose tissue) and ten that were more distant (e.g., liver and pituitary gland). We formed multiple sub-cohorts for each tissue by choosing 15 samples at random and then compared the results of HBA-DEALS between different sub-cohorts. Although the individual samples derive from unrelated individuals, there was a high degree of overlap of genes identified as differentially expressed or spliced. We tested the overlap between a total of 2791 pairs of cohorts (Additional file 1: Table S1). In each case, the overlap was highly significant (*p*<2.23×10^−308^ for all comparisons, hypergeometric test). These results suggest that HBA-DEALS is able to identify characteristic and reproducible differences in cohorts (Fig. 2c, d and Additional file 1: Figs. S4 and S5). The correlation was higher in the distant tissues, likely because there were more pronounced differences between samples. We also verified that the correlation increases with cohort size (Additional file 1: Fig. S6).

A runtime analysis showed that HBA-DEALS required roughly 1.2 h on 64 cores to perform 100,000 MCMC steps plus 10,000 warmup steps, which was roughly three times the time required by rMATS to analyze splicing events. With the exception of baySeq, which required 11 min, the other programs for the analysis of differential expression all finished within 1 min (Additional file 1: Fig. S7). The length of the Markov chain required for accurate estimation of the posterior and hence the total running time may be shorter in practice depending on dataset properties.

Finally, for each tissue pair, we performed multidimensional scaling (MDS) of the vectors of isoform proportions in all available samples, including only isoforms that were differentially spliced in at least 3 sub-cohorts (the “Methods” section). More specifically, we first obtained genes that were predicted to be differentially spliced in at least 3 cohorts of a given tissue pair, and that had isoforms that were classified as differentially spliced with a fold change of at least 2. For each gene, we then computed the proportion of each isoform that was predicted to be differentially spliced out of the total number of isoforms of that gene. The Euclidean distance between the vectors of these proportions over all differentially spliced genes of a tissue pair was then reduced into 2 dimensions using multidimensional scaling [25]. For example, MDS analysis of intersample distance based on levels of isoforms that had been identified as differentially spliced in different cohorts obtained a nearly perfect separation on samples from the left ventricle of the heart and the atrial appendage. As a control, we repeated the MDS with differentially expressed genes and isoforms that were assigned a probability of at least 0.25, and the two cohorts display a substantially lower degree of separation (Additional file 1: Fig. S8b).

### HBA-DEALS defines four categories of genes that differ with respect to splicing and expression

We next asked whether sets of genes can be identified by HBA-DEALS whose regulation is found to occur primarily by means of differential splicing, differential expression, or both. We performed 20 comparisons between samples from different tissues (e.g., left ventricle against atrial appendage). For each comparison, we compared cohorts of 15 samples for each tissue (Additional file 1: Table S1) and used HBA-DEALS to call genes differentially spliced, differentially expressed, both, or neither.

**Fig. 3:**
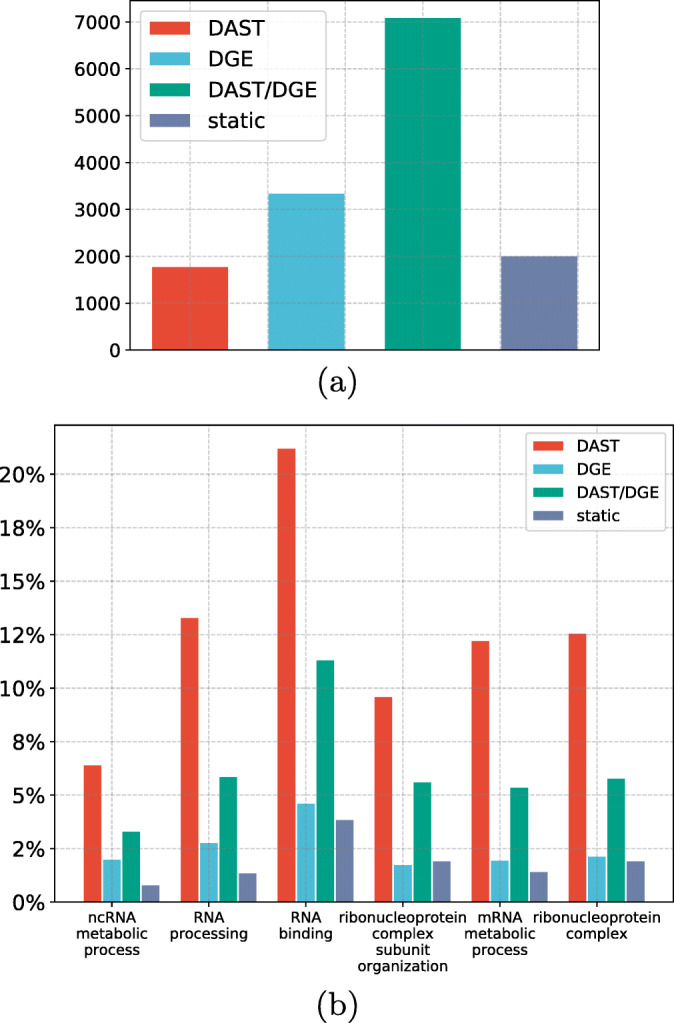
**a** Distribution of protein coding genes in the groups DAST (*n* = 1789), DGE (*n* = 3355), DAST/DGE (*n* = 7102), and static (*n* = 2018). Only those genes were included in the analysis for which at least two isoforms were expressed at sufficient depth in most tissues. **b** Gene Ontology analysis revealed six GO significantly enriched GO terms that show an at least 2-fold enrichment in one of the four groups (the “Methods” section and Supplemental Tables S3-S8)

For the following analysis, a gene is considered differentially expressed in a tissue if it was differentially expressed in at least 3 sub-cohorts, and differentially spliced if it has an isoform that was differentially spliced in at least 3 sub-cohorts. If a gene is found to be differentially spliced in at least twice as many comparisons as it is found to be differentially expressed, we assign it to the DAST group. Conversely, if a gene is differentially expressed in at least twice as many comparisons as it is differentially spliced, we assign it to the DGE group. Genes that are both differentially spliced and differentially expressed are assigned to the DAST/DGE group. Finally, genes that do not display differential expression or splicing as defined above are assigned to the static group (Fig. 3a).

We performed Gene Ontology [26] (GO) term enrichment analysis on the genes in each of the four groups. The four groups differed with respect to significantly overrepresented GO terms (Fig. 3b, with details in Supplemental Tables S3-S8). We classified GO terms as having strong enrichment if they displayed statistically significant overrepresentation, had at least 20 annotated genes, and showed an at least two-fold higher percentage of annotated genes than the entire population of genes (the “Methods” section). Six enrichments were found for the DAST group, and all of the terms were related to RNA biology. No strong enrichments were found for the other three groups. For instance, while only 10.1% of all 13,688 genes were annotated to rna binding, over twice as many genes in the DAST set were (21.5%). RNA binding proteins are involved in each step of RNA metabolism including alternative splicing [27]. Changes in alternative splicing are common in biological processes and disease states, and an investigation of the functions of alternatively spliced genes may tell us something about the biology of those states. For example, subsets of alternatively spliced genes found in aging, with mutations in the spliceosome gene *U2AF1* in myelodysplastic syndrome and with differentiation of erythroblasts, are enriched for genes involved in RNA processing [28–30].

None of the significant GO terms for the DGE group was associated with a two-fold increase in the percentage of annotated genes. The specificity of the significant GO terms identified for the DAST and DGE groups was significantly higher than that for the remaining two groups (Additional file 1: Fig. S9). This suggested that the DAST and DGE groups show a greater degree of functional uniformity than the other two groups, which motivated us to further investigate differences between these two groups.

### Pervasive differences in the genomic characteristics of DAST and DGE genes

We compared the DAST and DGE groups with respect to a variety of genomic properties. DAST genes show a lower percentage of promoter methylation than DGE genes. DAST genes also have a higher number of exons, are shorter, and have a lower mean exon length. DGE genes are more likely to have a TATA-box element in their promoter and are correspondingly less likely to be associated with a CpG island. DAST and DGE genes differ with respect to the frequency of a number of predicted transcription factor binding sites (Table 1).

**Table 1:** Genes regulated predominantly by alternative splicing (AS genes) differ from genes regulated predominantly by differential expression (DE genes) with respect to a number of characteristics related to DNA sequence, methylation, and transcription factor binding. Entries shown as percentages indicate the percentage of all genes in the group that display the characteristic. *MW* Mann-Whitney test, *FET* Fisher exact test, *W* Wald test. Additional methylation results are listed in Supplementary Table S9

Predictor	DAST genes	DGE genes	*p* value
Promoter methylation age 0	34.66	25.09	1.69×10^−23^ (MW)
ZFX binding	57.5%	35.8%	1.83×10^−8^ (W)
Number of exons	34	21	4.22×10^−106^ (MW)
Total gene length	72,367 bp	76,408 bp	5.81×10^−6^ (MW)
Dispersion	27.32	27.92	6.62×10^−3^ (MW)
CpG island	77%	61.3%	1.52×10^−26^ (FET)
TATA box	5.5%	12.9%	2.32×10^−15^ (FET)
Number of transcripts	11.4	6.9	4.94×10^−152^ (MW)
Exon length	240 bp	250 bp	4.35×10^−28^ (MW)

**Fig. 4:**
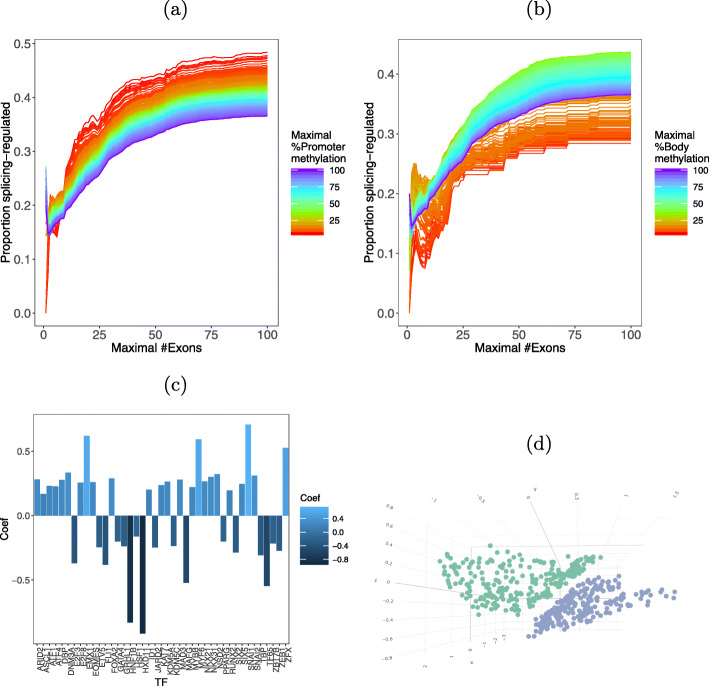
**a** Methylation × number of exons × proportion of splicing genes—promoter. **b** Methylation × number of exons × proportion of splicing genes—gene body. **c** Bar plot of the 41 TFs with significant regression coefficients in the logistic regresssion. Positive coefficients predict DAST, and negative coefficients predict DGE. **d** Multidimensional scaling of TF interaction profiles of DAST (blue) and DGE (green) genes

DNA methylation of the promoter region or of the gene body can influence alternative splicing [31–33]. We compared the methylation levels in gene body and promoter of DAST and DGE genes, in 12 different age groups (the “Methods” section). In all the age groups, the percentage of methylation was significantly higher in expression-regulated genes. We found that the degree of promoter and gene body methylation is also correlated with the exon count of genes. The proportion of DAST genes increases with the exon count, but for any particular exon count, lower degrees of promoter methylation are associated with higher proportions of DAST genes (Fig. 4a). For gene body methylation, the proportion of DAST genes is highest with intermediate levels of methylation and lowest with the lowest levels of methylation (Fig. 4b).

Limited evidence exists coupling binding of transcription factors to promoters with alternative splicing [34, 35]. We explored whether profiles of TF binding motifs in gene promoters are predictive of a gene being in the DAST or DGE group by means of logistic regression with a total of 401 predictors consisting of the predicted target genes of 401 TFs. The value of each predictor is 1 (TFBS present) or 0 (no TFBS). The dependent variable is the group (DAST vs. DGE). The weighted sum of the predictors and the intercept models the logit of the probability of belonging to the expression-regulated gene class. The model identified 41 TFs with statistically significant regression coefficients, comprising 24 TFs in the DAST group and 17 TFs in the DGE group (Fig. 4c and Additional file 1: Table S10).

### Networks of splicing-regulated transcription factors

We then investigated potential synergy between TFs of the DAST and DGE groups. For each group and for each pair of TFs, we computed the number of targets bound by both TFs divided by the number of targets bound by at least one of them, where targets are defined as all genes in either the DAST or the DGE groups. We noted that some TFs had very few or very many binding targets. In order to remove trivial low and high scores, we therefore selected TFs from the 0.2 to the 0.8 quantiles with respect to the total number of targets in both sets of genes. We then performed multidimensional scaling on the vectors of interaction scores of the different TFs, i.e., each point in the MDS corresponds to the interaction profile of a specific TF with other TFs when the targets are either the DAST or DGE set (Fig. 4d). Remarkably, we obtained perfect linear separation between interaction profiles for the two types of genes. This result suggests that combinatorial regulation plays a role in determining both changes in splicing and gene expression.

In order to examine whether particular protein complexes are enriched for DAST, we downloaded the full set of gene complexes from the CORUM database [36] and computed the probability of obtaining the observed proportion of DAST genes or a higher proportion under the binomial null distribution, setting the probability of a DAST gene to the mean proportion of DAST genes over all complexes (0.55). After Benjamini-Hochberg multiple testing correction, DAST but not DGE were significantly enriched in spliceosome-related complexes. Two complexes enriched for DAST genes were related to the ribosome (Table 2). Interestingly, networks of autoregulated alternative pre-mRNA splicing have been demonstrated for components of the spliceosome as well as for subsets of ribosomal proteins [37–39].

**Table 2:** Enrichment of DAST genes in protein complexes was calculated using the binomial distribution (the “Methods” section). The Benjamini-Hochberg-corrected *p* value is shown

Complex name	DAST genes	DGE genes	*p* value
Spliceosome	49	6	1.79×10^−6^
Nop56p-associated pre-rRNA complex	30	2	5.23×10^−5^
Ribosome, cytoplasmic	25	1	1.13×10^−4^
C complex spliceosome	32	4	4.08×10^−4^
60S ribosomal subunit, cytoplasmic	16	0	1.87×10^−3^
Parvulin-associated pre-rRNP complex	17	2	0.040

Combinatorial interactions among TFs and TF subnetworks are critical for tissue-specific gene expression [40]. We therefore asked to what extent transcription factors represented in the DAST and DGE groups differ. We computed the count of DAST-TFs with TF binding motifs in promoters of all DAST genes and the corresponding count with motifs in promoters or DGE genes. There was a significantly higher count for DAST genes as compared to DGE genes (16.05 vs. 13.67 binding TFs, *p*=7.36×10^−53^, Mann-Whitney test). This finding supports the possible existence of independent cellular circuits that are based primarily on changes in alternative splicing (Additional file 1: Fig. S10).

## Discussion

Although short- and long-read RNA sequencing has become a standard method for characterizing both gene expression and alternative splicing, the interplay between gene expression and alternative splicing has not been extensively studied. The majority of existing methods interrogate either gene expression or alternative splicing but not both, and methods for jointly modeling expression and splicing have been lacking. In this work, we have presented HBA-DEALS, a Bayesian method that analyzes both splicing and expression in a single model. Using simulated data, we have shown that the performance of HBA-DEALS in the identification of differential gene expression and differential alternative splicing is superior to that of state-of-the-art approaches. By investigating data of the GTEx project, we additionally showed that the predictions of HBA-DEALS are reproducible across independent biological cohorts. The algorithmic approach HBA-DEALS is a paradigm that can be used to identify groups of genes that display DAST, DGE, both, or neither. This type of analysis has not been readily available with existing methods that investigate DGE or DAST separately. We have demonstrated the utility of our method by investigating patterns of differential expression and splicing in different tissue types available in the GTEx resource. While both expression and splicing are controlled by a broad range of interacting regulatory signals [41–43], subsets of genes can be identified whose regulation occurs predominantly through alternative splicing (DAST) or through differential gene expression (DGE). To demonstrate a typical application of our algorithm, we characterized a set of genes that are preferentially alternatively spliced over a large number of comparisons of different tissues using data from the GTEx project. We characterized a set of genes that are preferentially alternatively spliced across comparisons of 20 tissue types. The DAST set was enriched in functions related to RNA metabolism, for members of spliceosomal and ribosomal protein complexes, and showed pervasive differences compared to the DGE set with respect to gene structure (gene length, average exon length, total exon count), DNA methylation of both promoter and gene body sequences, and the distribution of transcription factor binding sites. We have further found that combinatorial regulation of genes by transcription factors is fundamentally different in splicing-regulated and expression-regulated genes, which suggests that both processes are under the control of different gene regulatory networks. It seems plausible that each process can act independently under certain conditions or given a specific set of triggers. In support of this hypothesis, we found that transcription factors that are themselves in the DAST group favor targets that are in the DAST group. Moreover, DAST genes are enriched for RNA binding, suggesting possible post-transcriptional regulatory interactions.

## Conclusions

HBA-DEALS presents a novel paradigm for analyzing high-throughput transcriptional data. In contrast to previous approaches, the model used by HBA-DEALS includes both changes in gene expression levels and isoform proportions, thereby unifying aspects of transcriptional regulation that have thus far been analyzed separately. This level of analysis is paramount for understanding how the different levels of transcriptional regulation interact. Differential expression and alternative splicing have both been the focus of numerous studies that make use of high-throughput technologies. The unified approach to transcriptional modeling that we presented here is expected to improve the insights obtained from such studies by revealing the regulatory pathways that are triggered under different conditions or biological states.

## Methods

### HBA-DEALS: Hierarchical Bayesian Analysis of Differential Expression and ALternative Splicing

Hierarchical Bayesian modeling (HBM) is a multiparameter modeling technique in which one assumes a statistical distribution for individual parameters whose interdependencies are reflected in the structure of the hierarchy. In HBA-DEALS, this hierarchy models isoform levels as fractions of the total number of mRNA molecules produced from a certain gene. Our assumptions in developing our model were as follows: (i) An increase or decrease in gene expression induces an increase or decrease in the level of at least one isoform; (ii) Isoform levels are fractions of the gene expression level; and (iii) Changes in isoform fractions do not necessitate changes in expression levels and vice versa. A Markov Chain Monte-Carlo (MCMC) technique can be used to estimate the posterior probability of the parameters of an HBM. To do so, one must design the structure of the HBM and define the probability distribution of each node.

The input for HBA-DEALS consists of a matrix of isoform counts derived from two different conditions, here referred to as *case* and *control*, using any short- or long-read next-generation sequencing technology. In the case of short-read RNA-seq, tools such as RSEM [17] or StringTie [19] can be used to calculate isoform counts. Long-read isoform counts can be generated with pipelines such as SQANTI [20]. HBA-DEALS calculates gene expression levels by summing up the isoform counts of individual genes.

The data is log-transformed using *log*_2_(*x*+0.5), where *x* is the count-per-million reads (log-cpm). Expression levels are modeled as Normal, with mean that is the log-transformed sum of the corresponding isoform levels, and sample-specific variance, $\hat {\sigma }_{i}^{2}$ that is obtained from a mean-variance trend by fitting a linear model to each gene’s levels, and then fitting a trend line to the square root standard deviations as a function of mean gene level [6].

### Assessment of DGE

HBA-DEALS models gene expression as follows. The difference between cases and controls (*β*) is modeled with a weakly informative Normal prior.

1 $$ \beta \sim \text{Normal}(\beta | 0,5)  $$

The mean log-cpm level in controls (*β*_0_) is modeled with a Normal distribution such that the mean of its prior is equal to the log-transformed mean expression value of the control samples (*μ*_1_) and the variance is equal to 5. This constitutes a weakly informative prior since it expresses our belief that the value of *β*_0_ will most likely be close to the observed mean, but allows for large deviations:

2 $$ \beta_{0} \sim \text{Normal}(\beta_{0} |\mu_{1},5)  $$

In order to model differences between cases and controls, we model the expression in control *i* (*y*_*i*_) as:

3 $$ y_{i} \sim \text{Normal}(y_{i} |\beta_{0},\hat{\sigma}_{i}^{2})  $$

For case *j*, the mean is defined as *β*_0_+*β*:

4 $$ y_{j} \sim \text{Normal}(y_{j} |\beta_{0} +\beta,\hat{\sigma}_{j}^{2})  $$

where $\hat {\sigma }_{i}^{2}, \hat {\sigma }_{j}^{2}$ are obtained from the mean-variance trend.

### Assessment of DAST

Log-transformed isoform levels are also modeled as Normal, with variance that is obtained from a mean-variance trend, similarly to expression levels. The mean of isoform *i* corresponds to a fraction *p*_*i*_ of the mean expression level, with $\sum p_{i} = 1$.

The control fractions have a *Dirichlet*(1) prior, and the case fractions relate to the control fractions (*p*_*i*_ and $p_{i}^{\prime }$ refer to the proportion of isoform *i* in controls and cases) via the following formula (the Aitchison perturbation [21]):

5 $$ p_{i}^{\prime} = \frac{p_{i} \cdot \alpha_{i}}{\sum_{1 \leq j \leq T} p_{j} \cdot \alpha_{j}},  $$

where *T* is the number of isoforms of the gene and *α* is a vector whose entries sum to 1. This Aitchison perturbation computes the product of corresponding entries in the two vectors and divides each entry in the resulting vector by the sum of its entries. For example, if a gene has two isoforms with fractions (0.4,0.6) in condition 1, and *α* is (0.6,0.4), then the Aitchison perturbation will map the fraction of each isoforms to $\frac {0.4\cdot 0.6}{0.6\cdot 0.4+0.4\cdot 0.6}=0.5$ in condition 2. A vector whose entries are equal and sum to 1 is the identity element of the Abelian group defined on the simplex by the Aitchison perturbation. Therefore, a gene is not differentially spliced if and only if all the entries of *α* are equal. The prior on *α* is also set to Dirichlet with the vector $\overrightarrow {1}$. The Dirichlet prior is non-informative and is used in order not to make prior assumptions on isoform proportions and significant changes between cases and controls.

### Probability of differential expression/splicing

Our interpretation of whether a gene is differentially expressed is based on whether at least a certain proportion (in the GTEx cohort used in this paper, 99%) of the marginal posterior distribution of *β* is either above or below zero (if a gene is differentially expressed and that proportion is positive, cases are upregulated compared to controls; if negative, cases are downregulated compared to controls). Specifically, we sum the marginal probability of *β* over values that have the same sign as its mean.

In order to assess differential splicing, we examine $P_{\alpha _{i}}(x)$, defined as the marginal posterior of *α* for isoform *i*. If there is no differential splicing, then $\alpha =[\frac {1}{T},\frac {1}{T},...,\frac {1}{T}]$, where *T* is the number of isoforms. To determine whether a gene is differentially spliced, we examine the proportion of the distribution $P_{\alpha _{i}}(x-\frac {1}{T})$ that is above or below zero. If the bulk of the distribution (we used a threshold 99% for the analyses reported in this work) is above zero, then isoform *i* is upregulated in cases compared to controls, and vice versa. For the determination of gene-level differential splicing (i.e., deciding whether one or more isoforms is differentially spliced), the maximum probability over all isoforms is reported.

HBA-DEALS can also perform specific isoform-level analysis by setting the isoform.level parameter to true. We first note that if there is no differential splicing, the dot product between *α* and *p* (the vector of frequencies of individual isoforms in controls) is $\alpha \cdot p = \frac {1}{T}$. This is not true in general, and if there is differential splicing, *α*·*p* can be greater or less than $\frac {1}{T}$. HBA-DEALS calculates the probability that the *i*th isoform is differentially spliced by assessing the proportion of the distribution $P_{\alpha _{i}} (x-\alpha \cdot p)$ that is above (more of the *i*th isoform is present in controls) or below (less of the *i*th isoform is present in controls) zero. Again, if the bulk of the distribution (99.9% for the analysis reported here) is above zero, then isoform *i* is upregulated in cases compared to controls, and vice versa.

For the comparisons shown in Fig. 2b, isoform-level analysis was performed. For the remaining analyses, gene-level analysis of differential splicing was performed.

Finally, we classify a gene as DGE if the probability obtained for *β* as described above was above the probability threshold for differential expression and the probability obtained for *α* did not pass the probability threshold for differential splicing. We classify a gene as DAST if the probability obtained for *β* was not above the probability threshold for differential expression and the probability obtained for *α* was above the probability threshold for differential splicing. HBA-DEALS reports the mean posterior probabilities and the user can choose thresholds appropriate to the analysis.

### MCMC

We used the stan package via its R interface rstan [44] for finding the posterior probabilities. For the GTEx dataset, we set the number of chains to 1, the number of warmup steps to 2000, and the number of total steps to 10000. For the simulation, we set the number of steps to 100000, and the number of warmup iterations to 10000. We used the R package coda to parse the results. For MCMC initialization, gene expression values are set to the log-transformed sum of isoform counts, *β* is set to 0, isoform fractions in controls are set to the observed fractions $p_{i}=\frac {2^{tr_{i}}}{\sum _{1\leq j\leq T}2^{tr_{j}}}$ where *tr*_*i*_ is the log-cpm level of the *i*th isoform, and *α*’s entries are set to uniform values that sum to 1, i.e., $\alpha _{i}=\frac {1}{T}$ where *T* is the number of isoforms. All other parameters for stan were the defaults defined by rstan, including the No-U-Turn Sampler (NUTS).

### Optimal splicing using the t-statistic and proportions (OSTP)

In each sample, we divided the level of each isoform by the sum of levels of the other isoforms to obtain isoform proportions. For each proportion of false positives (FP), we found the t-statistic that maximizes the proportion of true positives (TP) in each simulated dataset, where the proportion of TPs is the proportion of differentially spliced isoforms with a fold change of 2 or greater that are detected. We here refer to the results obtained in this way as Optimal splicing using the t-statistic and proportions, or OSTP.

### GTEx dataset

The genome-wide, cross-tissue expression profiling provided by GTEx includes over 17 thousand expression samples from 948 donors in 54 tissues [15]. RSEM [17] isoform counts were extracted from the file GTEx_Analysis_2016-01-15_v7_RSEMv1.2.22_transcript_expected_count.txt, which is available from the GTEx portal [15]. This file contains RSEM counts for multiple isoforms of different genes identified by Ensembl IDs, in different tissues of different donors. We used biomaRt to map Ensembl IDs to HGNC gene symbols [45]. The sample annotations were extracted from the file GTEx_v7_Annotations_SampleAttributesDS.txt, which contains data from 8444 samples from 703 donors.

### Robustness analysis of HBA-DEALS

We reasoned that if HBA-DEALS is able to robustly identify mRNAs that are consistently differentially expressed, alternatively spliced, both, or neither, then we should observe a high level of consistency in its results for subsets of samples in the GTEx dataset. Therefore, for each pair of tissues, we randomly divided the data into sub-cohorts of 30 samples, 15 from each condition, keeping transcripts that had a count of at least 1 in each sub-cohort sample. We then ran HBA-DEALS on each sub-cohort separately and compared the sets of genes and isoforms that were identified as differentially expressed and differentially spliced, respectively. There was a highly significant overlap amongst both genes and isoforms that were consistently identified between cohorts. We then compared the changes in gene expression levels and isoform proportions quantitatively, by computing *R*^2^ for the log-fold expression changes of each gene and log-fold isoform proportion changes of each isoform. This resulted in high correlation between cohorts in the different tissues (Fig. 2c, d). As expected, tissues that were not related showed an overall higher correlation, since differences between tissues are much greater than differences between donors.

Fold changes in expression were calculated as the mean of the posterior of *β*, and fold changes in splicing as the Aitchison perturbation between the mean of the posterior of the fraction in controls and the mean of the posterior of *α* divided by the mean of the posterior of the fraction in controls.

To further determine the robustness of the set of isoforms that were identified as differentially spliced, we converted the GTEx expression data into isoform proportions and selected the set of isoforms that were differentially spliced in at least 3 cohorts and had a fold change of at least 2 for a multidimensional scaling of all the samples together. We used the R function cmdscale. The clear separation between samples belonging to different tissues confirmed the robustness of the isoforms identified in individual cohorts and their consistency as a set. In order to validate the visual observation, we computed the ratio of the mean between-tissue-distances to within-tissue-distances in the MDS for the real data and for 1000 permutations of the tissue labels, for each pair of tissues. We then counted the number of times that a value computed for the permuted labels was greater or equal to the corresponding value computed for the original labels. For all 10 tissue pairs, this did not occur in any of the permutations, corresponding to a *p* value <0.001 that a separation between two labels occurred by chance.

### Multidimensional scaling

Multidimensional scaling (MDS) is a nonlinear transformation that translates a matrix of pairwise distances between objects into a two-dimensional visualization of the objects that preserves the pairwise distances as much as possible [25, 46].

### Gene Ontology analysis

For Gene Ontology enrichment analysis, we used the program Ontologizer [47], using the Parent-Child Intersection algorithm [48]. The population set was composed of all the genes that passed the minimum-counts threshold, i.e., that participated in the analysis. The complete lists of GO categories with Bonferroni-corrected *p* values less or equal to 0.01 are given in Additional file 1: Tables S5, S6, S7, and S8. We used the go.obo and goa_human.gaf files downloaded on December 2, 2019.

### Defining four mRNA categories

We define splicing-regulated genes as genes for which differential splicing was observed at least twice as often as differential expression, and expression-regulated genes as genes for which differential expression was observed at least twice as often as differential splicing of one of the isoforms.

### Additional data sources

We have used several additional data sources in order to characterize the properties of splicing- and expression-regulated genes. Transcription start site (TSS) dispersion was obtained based on data from the FANTOM project portal [49]. For TF targets, we used the file hg38.gencode_v28.TF_HUMAN.tsv, where TF is the transcription factor name. Gene lengths were retrieved using the biomaRt R package. Exon and isoform annotations were taken from the file Homo_sapiens.GRCh38.91.gtf that was download from the Ensembl website. The methylation datasets were downloaded from MethBank [50]. The age groups were age0, age2-4, age5-13, age14-16, age17-28, age29-36, age37-42, age43-53, age54-66, age67-75, age76-88, and age89-101.

### Simulation

We used the code provided with [6] to simulate isoform levels and followed the methodology that was used to add differential expression for adding differential splicing. For each gene, a number of expressed isoforms was randomly generated between 2 and 10 using the probabilities 0.4,0.2,0.1,0.05,0.05,0.05,0.05,0.05,0.05. The proportions of genes were then divided by the corresponding number of isoforms. A set of differentially spliced genes was selected at random. In the original code, a gene’s proportion is multiplied by 2 in either cases or controls to generate differential expression. Therefore, for each differentially spliced gene, the proportion of one random isoform was increased 2-fold in cases, and the proportion of another isoform was increased 2-fold in controls. This ensures that the total proportion of the gene remains unchanged. After generating isoform proportions, the simulation proceeds as the original code. We generated datasets with random seeds 1–50. The first 25 were generated using equal library sizes and the last 25 with unequal library sizes. The input to rMATS consists of counts for “skip” and “inclusion”, each representing a distinct isoform. We set for each isoform the “skip” count as the number of counts of the isoform, and the “inclusion” count the number of counts of the other isoforms. We set isoform length to 1 and the PSI cutoff to 1e −10. Tools provided as R packages were used according to the usage instructions in the packages. In order to generate mean precision-recall curves, we fitted a trendline with the function lowess in the R package limma to the precision and recall values for each tool in each dataset. For missing precision values, we added points with the nearest lower recall value. The mean of the trendline values over all the datasets was then computed for each tool to obtain the mean precision-recall curve. For the precision-recall plots in Fig. 2a-b, 1-precision (FDR) is measured for statistic values between 0 and 1 in intervals of 10^−4^ for both expression and splicing.

The following command generates a simulated dataset using the HBA-DEALS R package: hbadeals::simulate(rseed=1). For this manuscript, 50 simulated datasets were generated using seeds 1 to 50.

### Statistical tests

For performing the Mann-Whitney test and Fisher’s exact test, creating the logistic regression model, computing the hypergeometric cdf, the t-statistic, and the Mann-Whitney statistic, we used the core modules from the R programming language version 3.4.1.

### Dispersion

Promoters can be characterized as either sharp type or broad type, depending on whether they contain one dominant transcription start site or multiple transcription start sites [51]. Cap analysis of gene expression (CAGE) can be used to identify transcription start sites in promoters. CAGE experiments generate sets of 20- to 27-bp sequence tags from the 5 ^′^ ends of mRNA, which can be matched to a reference genome. Any accumulation of tags (“peak”) is a reliable indicator of a transcription start site.

Based on FANTOM5 data [52], we computed dispersion indexes of CAGE tags for all promoter sequences, a metric that is conceptually similar to the standard deviation of tag counts [53]. A low dispersion index indicates a sharp distribution of tags, and a high dispersion index indicates a broad distribution of tags. To compute dispersion indexes, we counted tags between positions − 100 and + 100 relative to and on the same strand as the annotated transcription start sites. Let *s* be the dispersion index and *x*_*i*_ be the number of tags at position *i*. Then,

$$\begin{array}{*{20}l} c = \sum_{i=-100}^{100}{x_{i}} \qquad m = \frac{1}{c}\sum_{i=-100}^{100}{x_{i} i} \qquad s = \sqrt{\frac{1}{c}\sum_{i=-100}^{100}{x_{i}(i-m)^{2}}} \end{array} $$

The significance of the difference between the DAST and the DGE groups was determined using the Mann-Whitney test.

### CpG islands

In the human genome, CpG dinucleotides are present at about 20% of the frequency that would be expected based on the overall GC content. The depletion of CpG dinucleotides in the human and other mammalian genomes is due to the increased mutability of methylcytosine within CpG dinucleotides. Stretches of GC-rich (∼ 65%) sequence in which the observed frequency of CpG dinucleotides is close to the frequency that would be expected based on the individual frequency of G and C bases are termed CpG islands (CGIs). CGIs are associated with the upstream region of many genes generally covering all or part of the promoter and typically display an average size of about 1 kb [54, 55].

To identify CGIs in this study, a 100-nucleotide window was shifted in 1-bp intervals across the promoter sequences from position [−200,−100) relative to the TSS to (+100,+200]. The percentage GC content and CpG observed/expected ratio

$\frac {\mathrm {Number of CpG}}{\mathrm {Number of C}\times \mathrm {Number of G}}\times 100$

were calculated per window.

A promoter was considered having a CGI if the consecutive windows inside any region spanning at least 200 nt all had GC contents ≥50*%* and CpG observed/expected ratios ≥0.6 [56].

The significance of the difference between the DAST and DGE groups was determined using the Mann-Whitney test.

### TATA box

TATA boxes were identified as described [57].

### Number of isoforms

The number of isoforms per gene was retrieved from the GTF file Homo_sapiens.GRCh38.91.gtf. The significance of the difference between the DAST and DGE groups was determined using the Mann-Whitney test.

### Exon length

Exon lengths were retrieved from the GTF file +Homo_sapiens.GRCh38.91.gtf+. Overall gene length was retrieved from biomaRt [45]. The significance of the difference between the DAST and DGE groups was determined using the Mann-Whitney test.

### Software

HBA-DEALS is implemented as an R package that is freely available under the GNU General Public License version 3 (GPL3) at https://github.com/TheJacksonLaboratory/HBA-DEALS.

## Supplementary information

**Additional file 1** Supplemental material with figures S1-S10 and tables S1-S10.

**Additional file 2** Review history.

## References

[1] Pollard MO, Gurdasani D, Mentzer AJ, Porter T, Sandhu MS (2018). Long reads: their purpose and place. Hum Mol Genet.

[2] Wang Z, Gerstein M, Snyder M (2009). RNA-seq: a revolutionary tool for transcriptomics. Nat Rev Genet.

[3] Stark R, Grzelak M, Hadfield J (2019). RNA sequencing: the teenage years. Nat Rev Genet.

[4] Robinson MD, McCarthy DJ, Smyth GK (2010). edgeR: a Bioconductor package for differential expression analysis of digital gene expression data. Bioinformatics (Oxford, England).

[5] Love MI, Huber W, Anders S (2014). Moderated estimation of fold change and dispersion for RNA-seq data with DESeq2,. Genome Biol.

[6] Law CW, Chen Y, Shi W, Smyth GK (2014). voom: Precision weights unlock linear model analysis tools for RNA-seq read counts. Genome Biol.

[7] Sterne-Weiler T, Weatheritt RJ, Best AJ, Ha KCH, Blencowe BJ (2018). Efficient and accurate quantitative profiling of alternative splicing patterns of any complexity on a laptop. Mol Cell.

[8] Shen S, Park JW, Lu Z-x, Lin L, Henry MD, Wu YN, Zhou Q, Xing Y (2014). rMATS: robust and flexible detection of differential alternative splicing from replicate RNA-Seq data. Proc Nat Acad Sci U S A.

[9] Katz Y, Wang ET, Airoldi EM, Burge CB (2010). Analysis and design of RNA sequencing experiments for identifying isoform regulation. Nat Methods.

[10] Hu Y, Huang Y, Du Y, Orellana CF, Singh D, Johnson AR, Monroy A, Kuan P-F, Hammond SM, Makowski L, Randell SH, Chiang DY, Hayes DN, Jones C, Liu Y, Prins JF, Liu J (2012). DiffSplice: the genome-wide detection of differential splicing events with RNA-seq. Nucleic Acids Res.

[11] Sebestyén E, Zawisza M, Eyras E (2015). Detection of recurrent alternative splicing switches in tumor samples reveals novel signatures of cancer. Nucleic Acids Res.

[12] Kahles A, Ong CS, Zhong Y, Rätsch G (2016). SplAdder: identification, quantification and testing of alternative splicing events from RNA-seq data. Bioinformatics.

[13] Climente-González H, Porta-Pardo E, Godzik A, Eyras E (2017). The functional impact of alternative splicing in cancer. Cell Rep.

[14] Oshlack A, Wakefield MJ (2009). Transcript length bias in RNA-seq data confounds systems biology. Biol Direct.

[15] GTEx Consortium (2013). The genotype-tissue expression (GTEx) project. Nat Genet.

[16] Patro R, Duggal G, Love MI, Irizarry RA, Kingsford C (2017). Salmon provides fast and bias-aware quantification of transcript expression. Nat Methods.

[17] Li B, Dewey CN (2011). RSEM: accurate transcript quantification from RNA-seq data with or without a reference genome. BMC bioinformatics.

[18] Bray NL, Pimentel H, Melsted P, Pachter L (2016). Near-optimal probabilistic RNA-seq quantification. Nat Biotechnol.

[19] Pertea M, Pertea GM, Antonescu CM, Chang T-C, Mendell JT, Salzberg SL (2015). StringTie enables improved reconstruction of a transcriptome from RNA-seq reads. Nat Biotechnol.

[20] Tardaguila M, de la Fuente L, Marti C, Pereira C, Pardo-Palacios FJ, Del Risco H, Ferrell M, Mellado M, Macchietto M, Verheggen K, Edelmann M, Ezkurdia I, Vazquez J, Tress M, Mortazavi A, Martens L, Rodriguez-Navarro S, Moreno-Manzano V, Conesa A (2018). SQANTI: extensive characterization of long-read transcript sequences for quality control in full-length transcriptome identification and quantification. Genome Res.

[21] Aitchison J. The statistical analysis of compositional data: Springer Netherlands; 1986. 10.1007/978-94-009-4109-0.

[22] Geweke J, Bernado JM, Berger JO, Dawid AP, Smith AFM (1992). Evaluating the accuracy of sampling-based approaches to the calculation of posterior moments. Bayesian statistics 4.

[23] Hardcastle TJ, Kelly KA (2010). baySeq: empirical Bayesian methods for identifying differential expression in sequence count data. BMC bioinformatics.

[24] Tarazona S, Furió-Tarí P, Turrà D, Pietro AD, Nueda MJ, Ferrer A, Conesa A (2015). Data quality aware analysis of differential expression in RNA-seq with NOISeq R/Bioc package. Nucleic Acids Res.

[25] Mardia KV (1978). Some properties of clasical multi-dimesional scaling. Commun Stat Theory Methods.

[26] The Gene Ontology Consortium (2017). Expansion of the Gene Ontology knowledgebase and resources. Nucleic Acids Res.

[27] Fu X-D, Ares M (2014). Context-dependent control of alternative splicing by RNA-binding proteins. Nat Rev Genet.

[28] Pimentel H, Parra M, Gee SL, Mohandas N, Pachter L, Conboy JG (2016). A dynamic intron retention program enriched in rna processing genes regulates gene expression during terminal erythropoiesis. Nucleic Acids Res.

[29] Rodríguez SA, Grochová D, McKenna T, Borate B, Trivedi NS, Erdos MR, Eriksson M (2016). Global genome splicing analysis reveals an increased number of alternatively spliced genes with aging. Aging cell.

[30] Shirai CL, Ley JN, White BS, Kim S, Tibbitts J, Shao J, Ndonwi M, Wadugu B, Duncavage EJ, Okeyo-Owuor T, Liu T, Griffith M, McGrath S, Magrini V, Fulton RS, Fronick C, O’Laughlin M, Graubert TA, Walter MJ (2015). Mutant U2AF1 expression alters hematopoiesis and pre-mRNA splicing in vivo. Cancer cell.

[31] Young JI, Hong EP, Castle JC, Crespo-Barreto J, Bowman AB, Rose MF, Kang D, Richman R, Johnson JM, Berget S, Zoghbi HY (2005). Regulation of RNA splicing by the methylation-dependent transcriptional repressor methyl-CpG binding protein 2. Proc Nat Acad Sci U S A.

[32] Shukla S, Kavak E, Gregory M, Imashimizu M, Shutinoski B, Kashlev M, Oberdoerffer P, Sandberg R, Oberdoerffer S (2011). CTCF-promoted RNA polymerase II pausing links DNA methylation to splicing. Nature.

[33] Lev Maor G, Yearim A, Ast G (2015). The alternative role of DNA methylation in splicing regulation. Trends Genetics TIG.

[34] Cramer P, Cáceres JF, Cazalla D, Kadener S, Muro AF, Baralle FE, Kornblihtt AR (1999). Coupling of transcription with alternative splicing: RNA pol II promoters modulate SF2/ASF and 9G8 effects on an exonic splicing enhancer. Mol Cell.

[35] Damgaard CK, Kahns S, Lykke-Andersen S, Nielsen AL, Jensen TH, Kjems J (2008). A 5’ splice site enhances the recruitment of basal transcription initiation factors in vivo. Mol Cell.

[36] Giurgiu M, Reinhard J, Brauner B, Dunger-Kaltenbach I, Fobo G, Frishman G, Montrone C, Ruepp A (2019). CORUM: the comprehensive resource of mammalian protein complexes—2019. Nucleic Acids Res.

[37] Malygin AA, Parakhnevitch NM, Ivanov AV, Eperon IC, Karpova GG (2007). Human ribosomal protein s13 regulates expression of its own gene at the splicing step by a feedback mechanism. Nucleic Acids Res.

[38] Takei S, Togo-Ohno M, Suzuki Y, Kuroyanagi H. Evolutionarily conserved autoregulation of alternative pre-mRNA splicing by ribosomal protein L10a. Nucleic Acids Res. 2016. 10.1093/nar/gkw152. 10.1093/nar/gkw152 PMC4937301 26961311

[39] Lareau LF, Brenner SE (2015). Regulation of splicing factors by alternative splicing and NMD is conserved between kingdoms yet evolutionarily flexible. Mol Biol Evol.

[40] Ravasi T, Suzuki H, Cannistraci CV, Katayama S, Bajic VB, Tan K, Akalin A, Schmeier S, Kanamori-Katayama M, Bertin N, et. al (2010). An atlas of combinatorial transcriptional regulation in mouse and man. Cell.

[41] Louadi Z, Tayara H, Oubounyt M. Deep splicing code: classifying alternative splicing events using deep learning. Genes. 2019; 10. 10.3390/genes10080587. 10.3390/genes10080587 PMC6722613 31374967

[42] Bao S, Moakley DF, Zhang C (2019). The splicing code goes deep. Cell.

[43] Cramer P (2019). Organization and regulation of gene transcription. Nature.

[44] Carpenter B, Gelman A, Hoffman MD, Lee D, Goodrich B, Betancourt M, Brubaker M, Guo J, Li P, Riddell A. Stan: A probabilistic programming language. J Stat Softw. 2017; 76(1). 10.18637/jss.v076.i01. 10.18637/jss.v076.i01 PMC9788645 36568334

[45] Smedley D, Haider S, Ballester B, Holland R, London D, Thorisson G, Kasprzyk A (2009). Biomart–biological queries made easy. BMC genomics.

[46] Hout MC, Papesh MH, Goldinger SD (2013). Multidimensional scaling. Wiley Interdiscip Rev Cogn Sci.

[47] Bauer S, Grossmann S, Vingron M, Robinson PN (2008). Ontologizer 2.0–a multifunctional tool for GO term enrichment analysis and data exploration. Bioinformatics (Oxford, England).

[48] Grossmann S, Bauer S, Robinson PN, Vingron M (2007). Improved detection of overrepresentation of Gene-Ontology annotations with parent child analysis. Bioinformatics (Oxford, England).

[49] Noguchi S, Arakawa T, Fukuda S, Furuno M, Hasegawa A, Hori F, Ishikawa-Kato S, Kaida K, Kaiho A, Kanamori-Katayama M, et. al (2017). FANTOM5 CAGE profiles of human and mouse samples. Sci Data.

[50] Li R, Liang F, Li M, Zou D, Sun S, Zhao Y, Zhao W, Bao Y, Xiao J, Zhang Z (2018). MethBank 3.0: a database of DNA methylomes across a variety of species. Nucleic Acids Res.

[51] Carninci P, Sandelin A, Lenhard B, Katayama S, Shimokawa K, Ponjavic J, Semple CA, Taylor MS, Engström PG, Frith MC (2006). Genome-wide analysis of mammalian promoter architecture and evolution. Nat Genet.

[52] Arner E, Daub CO, Vitting-Seerup K, Andersson R, Lilje B, Drabløs F, Lennartsson A, Rönnerblad M, Hrydziuszko O, Vitezic M (2015). Transcribed enhancers lead waves of coordinated transcription in transitioning mammalian cells. Science.

[53] Dreos R, Ambrosini G, Bucher P (2016). Influence of rotational nucleosome positioning on transcription start site selection in animal promoters. PLoS Comput Biol.

[54] Larsen F, Gundersen G, Lopez R, Prydz H (1992). Cpg islands as gene markers in the human genome. Genomics.

[55] Robinson PN, Böhme U, Lopez R, Mundlos S, Nürnberg P (2004). Gene-Ontology analysis reveals association of tissue-specific 5’ CpG-island genes with development and embryogenesis. Hum Mol Genet.

[56] Gardiner-Garden M, Frommer M (1987). Cpg islands in vertebrate genomes. J Mol Biol.

[57] Steinhaus R, Gonzalez T, Seelow D, Robinson PN (2020). Pervasive and CpG-dependent promoter-like characteristics of transcribed enhancers. Nucleic Acids Res.

[58] Karlebach G, Robinson PN. Hierarchical Bayesian analysis of Differential Expression and ALternative Splicing (HBA-DEALS): GitHub repository. 2019. https://github.com/TheJacksonLaboratory/HBA-DEALS.

