# Supplementary material for: HBA-DEALS: accurate and simultaneous identification of differential expression and splicing using hierarchical Bayesian analysis
Source: Genome Biol. 2020 Jul 13;21:171. doi: 10.1186/s13059-020-02072-6 (PMC7358203; doi:10.1186/s13059-020-02072-6)
Supplement: Supplementary file 2 — Additional file 2 Review history. [file 13059_2020_2072_MOESM2_ESM.docx]

**1^st^ round**

**Reviewer 1**

The problem of jointly analyzing differential expression and alternative splicing is indeed important and the method to solve such a problem in an integrative analysis framework is intriguing.

As claimed by the authors, the proposed hierarchical Bayesian model should jointly model gene expression and isoform levels. However, based on what has been presented in the submission, it appears that the authors first preprocessed and normalized gene expression data and calculated isoform fractions. The modeling of gene expression and isoform fractions are based on Gaussian and Dirichlet assumptions. However, it is not clear to me how these two models are coupled so that differential expression and alternative splicing can be analyzed jointly as claimed.

There also seem to be several hyperparameters in HBA-DEALS, including variances in the prior distributions of beta's. It is not clear how these values were decided and whether the analysis results are sensitive to different choices.

There are many details should be provided, including 1) the MCMC inference procedure instead of simply stating using rstan package, 2) how the final differential expression and alternative splicing results were combined after checking the posterior distributions, 3) how different confounding factors, such as isoform lengths may affect the model and analysis results?

In summary, it appears that differential expression and alternative splicing analyses are still done separately. Clearer explanation on hyperparameter setups and inference procedures should be provided to allow replication and performance comparison.

**Reviewer 2**

The authors presented a new method which performs differential gene expression analysis on the isoforms, with the joint consideration of splicing variants and differential gene expression levels. They used a hierarchical Baysian framework with Dirichlet priors followed by log Normal distributions. The method is easy to understand, clearly written and seem to perform well. It was further applied to GTEx dataset and demonstrated reproducible calling of differentially expressed isoforms. Overall this referee finds this manuscript a potentially useful tool in RNA-seq analysis. There are several points that could be done to further strenghen the results:

Major:

1. It will be helpful for the authors to perform a computational time comparison between their and other methods. MCMC based approaches are usually slow, and it is unclear how it scales up to the genomics datasets, typically with 10^4-10^5 splicing variants to process.

2. Can this method be readily applied to single cell RNA-seq data, say, SMARTseq data with isoform calling? There are emerging datasets containing 10^5 cells generated in the public domain in the past few years, and the mainstream analysis focuses on overall gene expression levels. The utility of this method will be significantly expanded if it applies to single cell data as well.

3. The authors need to provide a schematic chart to illustrate their hierarchical Bayesian model.

4. Can the authors justify why a variance of 5 for the prior distribution of beta is considered non-informative?

5. This sentence in Methods is difficult to understand: "Therefore, we predict differential splicing of a specific isoform when the bulk of the marginal posterior density of the isoform's corresponding entry in α does not contain the scalar product of α and the vector of isoform proportion in condition 1.". First, is the scalar product of α and p is a constant or a variable? My understanding is that α is a variable that needs to be fit. If so, is it representing the fitted vector? Second, how to define "bulk"? The authors need to quantify this definition, i.e. 2 standard deviations away from the mean, etc.

6. Figure 2e-f: I am confused about the purpose of this analysis. The authors used differentially spliced isoforms to calculate intersample distance, and applied MDS to the distance matrix for 2-d visualization. The resulting scatter plot showed that the two samples being compared in the first place can be separated. Sure, they should. What is the purpose for this circular argument? To establish the cut-off for significant differential splicing calls?

Minor:

1. Somehow the table legends are inserted in the main body of the text, without proper delimiter, making it difficult to read.

2. It will be helpful for the authors to show that the MCMC chain is convergent given the parameters in the Methods, for genes with different number of isoforms.

**Author response**

Reviewer #1: The problem of jointly analyzing differential expression and alternative splicing is indeed important and the method to solve such a problem in an integrative analysis framework is intriguing.

1. As claimed by the authors, the proposed hierarchical Bayesian model should jointly model gene expression and isoform levels. However, based on what has been presented in the submission, it appears that the authors first preprocessed and normalized gene expression data and calculated isoform fractions. The modeling of gene expression and isoform fractions are based on Gaussian and Dirichlet assumptions. However, it is not clear to me how these two models are coupled so that differential expression and alternative splicing can be analyzed jointly as claimed.

Response: We thank the reviewer for pointing out the need for clarifying this and the following points in the manuscript. We have now revised the Results (page 2) and Methods (pages 10-11) sections in order to better explain how the hierarchical Bayesian model models both expression and splicing simultaneously, and added a supplementary figure that illustrates the different distributions used in the model (Additional file 1: Fig. S1).

(In the new text we have amplified the explanation and explicitly state: “Note that both α and β are parameters in a single hierarchical model, and therefore HBA-DEALS estimates the posterior distributions of α and β simultaneously”).

2. There also seem to be several hyperparameters in HBA-DEALS, including variances in the prior distributions of beta's. It is not clear how these values were decided and whether the analysis results are sensitive to different choices.

Response: We now discuss the choice of priors in the results in both the Results and Methods sections. The results are robust to different parameter values. For instance, we repeated the simulation using a different value of , and obtained essentially the same results, and the same for 0(Additional file 1: Fig. S2a-d).

3. There are many details should be provided, including 1) the MCMC inference procedure instead of simply stating using rstan package, 2) how the final differential expression and alternative splicing results were combined after checking the posterior distributions, 3) how different confounding factors, such as isoform lengths may affect the model and analysis results?

Response: We revised two subsections in the Methods section, “Probability of differential expression/splicing” and “MCMC”, in order to explain these points. Regarding isoform lengths, these are not expected to profoundly affect our results since RSEM takes isoform length into account in its predictions, and since as in the case of differential expression we are only interested in relative changes between controls and cases. It is not straightforward to prove this assertion as a gold standard across the scale of GTEx is not available.

4. In summary, it appears that differential expression and alternative splicing analyses are still done separately. Clearer explanation on hyperparameter setups and inference procedures should be provided to allow replication and performance comparison.

Response: We have revised the description of the method in the Results and in the Methods in order to increase clarity. See also our answer to comment 1.

Reviewer #2: GBIO-D-20-00043

The authors presented a new method which performs differential gene expression analysis on the isoforms, with the joint consideration of splicing variants and differential gene expression levels. They used a hierarchical Baysian framework with Dirichlet priors followed by log Normal distributions. The method is easy to understand, clearly written and seem to perform well. It was further applied to GTEx dataset and demonstrated reproducible calling of differentially expressed isoforms. Overall this referee finds this manuscript a potentially useful tool in RNA-seq analysis. There are several points that could be done to further strengthen the results:

Major:

1. It will be helpful for the authors to perform a computational time comparison between their and other methods. MCMC based approaches are usually slow, and it is unclear how it scales up to the genomics datasets, typically with 10^4-10^5 splicing variants to process.

Response: We thank the reviewer for bringing up the topic of running time which is indeed a very important aspect of the problem of modeling differential expression and splicing, and which we address in the revised version of the manuscript. The simulated datasets that we used contain a number of variants between 104-105 and so we used the running time that they require as the measure for comparison. Since rMATS and HBA-DEALS require the most computational resources and since both enable the use of multiple cores, we ran all the tools on our new research cluster, which includes 100 Supermicro BigTwin servers with 72 cores each at 2.7GHz and 768 GB of DDR4 RAM dedicating 64 cores per job. The running times are summarized in Supplementary Figure S7. As can be seen in the figure, HBA-DEALS has a somewhat longer running time, but it is not prohibitive using high-performance computing that is utilized in most projects involving next-generation sequencing.

2. Can this method be readily applied to single cell RNA-seq data, say, SMARTseq data with isoform calling? There are emerging datasets containing 10^5 cells generated in the public domain in the past few years, and the mainstream analysis focuses on overall gene expression levels. The utility of this method will be significantly expanded if it applies to single cell data as well.

Response: This is a great idea for extending the method. However, single-cell Pacbio RNA-seq data is not widely available, presumably because of the still high costs and the technical difficulties. We are aware of several posters about this on the PacBio website and of one colleague who is currently generating data with this method. However, we do not have access to sc Pacbio transcriptomic data. We also feel that it is likely that additional algorithmic steps will be required to deal with this data. For instance, zero-inflated statistics are required for some sc-RNA-seq algorithms using Illumina data. Therefore, we think that this goes beyond the scope of the current manuscript.

3. The authors need to provide a schematic chart to illustrate their hierarchical Bayesian model.

Response: In the revised version of the manuscript we provide a diagram that illustrates the model (Additional file 1: Figure S1), including all the distributions that the model uses.

4. Can the authors justify why a variance of 5 for the prior distribution of beta is considered non-informative?

Response: A variance of 5 in the prior distribution of beta takes into account a broad range of fold changes, however we agree that a “weakly-informative” prior would be more accurate here and we modified the text accordingly.

5. This sentence in Methods is difficult to understand: "Therefore, we predict differential splicing of a specific isoform when the bulk of the marginal posterior density of the isoform's corresponding entry in α does not contain the scalar product of α and the vector of isoform proportion in condition 1.". First, is the scalar product of α and p is a constant or a variable? My understanding is that α is a variable that needs to be fit. If so, is it representing the fitted vector? Second, how to define "bulk"? The authors need to quantify this definition, i.e. 2 standard deviations away from the mean, etc. Response: In order to clarify this sentence we have revised both the Methods section (pages 10-11) and the Results section (page 2).

6. Figure 2e-f: I am confused about the purpose of this analysis. The authors used differentially spliced isoforms to calculate intersample distance, and applied MDS to the distance matrix for 2-d visualization. The resulting scatter plot showed that the two samples being compared in the first place can be separated. Sure, they should. What is the purpose for this circular argument? To establish the cut-off for significant differential splicing calls?

Response: We have now moved frame e-f of figure 2 to the Additional file (Supplement). The MDS in this figure is applied to isoform proportions instead of isoform expression levels. Taken together, these proportions provide a strong enough signal to separate samples from the two tissues, or in other words samples from a specific tissue have a unique signature of isoform proportions.

Minor:

1. Somehow the table legends are inserted in the main body of the text, without proper delimiter, making it difficult to read.

Response: We apologize for this error which stemmed from problems in converting a latex template to pdf. We have now resolved this issue in the new version of the manuscript.

2. It will be helpful for the authors to show that the MCMC chain is convergent given the parameters in the Methods, for genes with different number of isoforms.

Response: This is a great point and we applied Geweke’s convergence diagnostic to the MCMC runs on a complete simulated dataset with 5000 warmup steps and 5000 MCMC steps. The null distribution is standard normal and after multiple testing correction none of the p-values obtained using Geweke’s diagnostic were significant. The p-values before and after correction are displayed in Additional file 1: Fig. S3a and S3b.

**2^nd^ round**

**Reviewer 1**

The new supplement figure to illustrate the connection of DGE and DAST models is appreciated. However, the details on inference procedure is still missing and method description should be improved significantly.

The detailed comments are below:

1. The supplement figure is appreciated. However, it is not clear how alpha and beta are coupled in the proposed model to lead to better DGE and DAST. As based on the presentation, beta is only for DGE while alpha for DAST and the connection seems to be based on the model of log "isoform levels" through expression levels. With this construct, it may help better illustrate the connection by writing out the detailed MCMC steps.

2. The presentation of methods (page 9 to 11) needs to be improved significantly, including math notations, confusing/imprecise statements, missing details, and typos. For example, in equations 3 and 4, variance sigma_i was never specified. The isoform level model was not given clearly in the main text if there is no Fig. S1, where the variance notation changed to delta, again not specified. Here are several confusing statements: 1. "Specifically, we sum the marginal probability of beta over values that have the same sign as its mean." 2. "we classify a gene as DGE if its ?value of beta? was above the probability threshold" (Did you check the posterior of beta to have 99% desired probability?) 3. "We used the stan package for MCMC ?modeling?" 4. "beta is set to 0," (are we supposed to infer beta posterior?) and many others. Last but not least, the notations of P_{\alpha_i}(x) and P_{\alpha_i}(x-alpha p) is very strange. Please write these representations in a mathematically correct way.

**Reviewer 2**

The authors have adequately addressed my concerns. As noted by the authors, the outstanding problem of this method is its computational time. DE analysis is typically a small, downstream part of the transcriptomics analysis pipeline. If the current method takes ~1.5 hours using 64 cores to complete, it may take more than 2 days on a personal computer with typically 1 core or less. Considering DE analysis might be done multiple times to compare different sample subsets, this computational burden might be prohibitively large. I think the authors should work on improving its computational efficiency in order to increase its utilities, as a future direction.

**Author response**

1. The new supplement figure to illustrate the connection of DGE and DAST models is

appreciated. However, the details on inference procedure is still missing and method

description should be improved significantly.

The supplement figure is appreciated. However, it is not clear how alpha and beta are

coupled in the proposed model to lead to better DGE and DAST. As based on the

presentation, beta is only for DGE while alpha for DAST and the connection seems to

be based on the model of log "isoform levels" through expression levels. With this

construct, it may help better illustrate the connection by writing out the detailed MCMC

steps.

=> We have revised the text to improve clarity of the methods description.

2. The presentation of methods (page 9 to 11) needs to be improved significantly,

including math notations, confusing/imprecise statements, missing details, and typos.

For example, in equations 3 and 4, variance sigma_i was never specified. The isoform

level model was not given clearly in the main text if there is no Fig. S1, where the

variance notation changed to delta, again not specified. Here are several confusing

statements: 1. "Specifically, we sum the marginal probability of beta over values that

have the same sign as its mean." 2. "we classify a gene as DGE if its ?value of beta?

was above the probability threshold" (Did you check the posterior of beta to have 99%

desired probability?) 3. "We used the stan package for MCMC ?modeling?" 4. "beta is

set to 0," (are we supposed to infer beta posterior?) and many others. Last but not

least, the notations of P_{\alpha_i}(x) and P_{\alpha_i}(x-alpha p) is very strange.

Please write these representations in a mathematically correct way.

=> We have added more explanations of this notation.

3. Reviewer #2: The authors have adequately addressed my concerns. As noted by

the authors, the outstanding problem of this method is its computational time. DE

analysis is typically a small, downstream part of the transcriptomics analysis pipeline. If

the current method takes ~1.5 hours using 64 cores to complete, it may take more than

2 days on a personal computer with typically 1 core or less. Considering DE analysis

might be done multiple times to compare different sample subsets, this computational

burden might be prohibitively large. I think the authors should work on improving its

computational efficiency in order to increase its utilities, as a future direction.

=> Our algorithm runs essentially overnight on a good laptop, but we suspect that most

users would run it on a cluster or cloud environment. It is an MCMC method, and thus

the accuracy depends on the number of steps, and the user can choose their desired

tradeoff. We have noticed minimal decreases in accuracy using half as many steps.

There are also some MCMC specific algorithmic approaches that might speed things

up somewhat. Currently, we think that the run time will be acceptable for real life use

cases, but if we do get complaints we will work on improving speed in the future.
